# Self‐Care Experiences of Psychiatric Nurses: A Phenomenological Study

**DOI:** 10.1111/jpm.70153

**Published:** 2026-05-22

**Authors:** Sibel Çaynak, Yeliz Karaçar, Buket Şimşek Arslan, Ahmet Göktaş, Halil İbrahim Karaçar

**Affiliations:** ^1^ Department of Nursing, Faculty of Health Sciences Antalya Bilim University Antalya Türkiye; ^2^ Department of Psychiatric Nursing, Faculty of Nursing Akdeniz University Antalya Türkiye; ^3^ Department of Nursing, Faculty of Health Sciences Burdur Mehmet Akif Ersoy University Burdur Türkiye; ^4^ Department of Nursing, Faculty of Health Sciences Bitlis Eren University Bitlis Türkiye; ^5^ Directorate of Health Care Services Antalya City Hospital Antalya Türkiye

**Keywords:** descriptive phenomenological study, mental health nursing, psychiatric nursing, self‐care

## Abstract

**Introduction:**

Self‐care is both a personal and professional responsibility for nurses; however, academic evidence in this area remains limited.

**Aim:**

Psychiatric nurses' self‐care experiences were examined.

**Method:**

This descriptive phenomenological study included 18 psychiatric nurses from a public hospital, using purposive sampling. Data were collected via semi‐structured, individual, and in‐depth face‐to‐face interviews conducted between May and June 2025. Analyses included Colaizzi's seven‐stage phenomenological analysis conducted using MAXQDA software.

**Results:**

Four themes and 13 subthemes were elicited. Nurses reported increased self‐care awareness, identified themselves as role models for patients, and emphasized the importance of physical, mental, social, spiritual, and professional self‐care, as well as daily routines. Facilitators and barriers included motivation, work‐life balance, and emotional burden as individual factors, and workplace experiences, shift work, and professional perspective as professional factors.

**Conclusion:**

Psychiatric nurses' self‐care awareness and strategies play a critical role in individual and professional well‐being and in the quality of care, underscoring the need for support at both individual and institutional levels.

**Implications for Practice:**

Training programs and institutional practices promoting self‐care awareness and work‐life balance may enhance nurses' well‐being and the quality of patient care.

**Relevance Statement:**

This study explored psychiatric nurses' self‐care experiences, highlighting factors that influence their well‐being and professional practice, including workplace dynamics, shift patterns, and role expectations. Findings on facilitators and barriers to self‐care provide valuable insights for designing educational programs and institutional strategies to support nurses' physical, mental, social, spiritual, and professional health. By addressing universal challenges, such as emotional burden and work‐life balance, while considering context‐specific factors in a specific healthcare context, this study contributes to enhancing psychiatric nursing practice and supporting nurses' well‐being in diverse settings.

## Accessible Summary

- What is known about the subject?
  ○ Self‐care is not only an individual necessity for nurses but also a fundamental component of professional responsibility and sustainable, high‐quality care.
  ○ Existing research has largely focused on the general nursing population; phenomenological studies that examine the self‐care experiences of psychiatric nurses in depth remain limited.
- What does this paper add to existing knowledge?
  ○ This study is one of the rare qualitative studies that provide in‐depth insights into psychiatric nurses' self‐care experiences using a phenomenological approach.
  ○ It identifies personal, professional, and organizational barriers to self‐care, as well as factors that facilitate it.
  ○ It highlights the diversity of physical, mental, social, spiritual, and professional self‐care strategies used by psychiatric nurses.
- What are the implications for practice?
  ○ Structured self‐care programs and training specifically designed for psychiatric nurses should be developed.
  ○ Institutional policies that support work‐life balance and provide social and professional support are needed.
  ○ The integration of stress management and self‐care strategies into both undergraduate nursing education and continuing professional development programs should be encouraged.
- What approach was taken to ensure the method used was good quality?
  ○ In‐depth interviews conducted by researchers with psychiatric nursing expertise ensured the collection of rich and meaningful descriptions of participants' lived experiences.
  ○ Reflexivity was maintained through acknowledgement of the researchers' preconceptions, memo writing, and team discussions, while collaboration with an independent qualitative expert enhanced analytical rigor.
  ○ Data were analysed using Colaizzi's seven‐step phenomenological method with MAXQDA software, guided by theoretical frameworks, and COREQ guidelines were followed to ensure methodological rigour and trustworthiness.

## 1 Introduction

Mental health and psychiatric nurses play a vital role in delivering care by addressing stigma and discrimination, promoting equitable access to services, and respecting individuals' dignity and autonomy (Stewart et al. 2024). In addition to these responsibilities, nurses are expected to maintain their own health, well‐being, professional integrity, and ethical standards (Linton and Koonmen 2020). Article 5 of the American Nurses Association (ANA) Code of Ethics frames nurses' self‐care not only as a personal need but also as an essential component of professional integrity and responsibility (American Nurses Association 2025). Similarly, the World Health Organization (WHO) recommends that health organizations implement interventions to support self‐care in order to enhance physical and mental well‐being and prevent burnout (World Health Organization 2022). Therefore, it is important to explore how the concept of self‐care is defined in mental health and psychiatric nursing practice, what dimensions it includes, and how nurses' self‐care experiences are shaped by it.

### 1.1 Background

Psychiatric nurses work in mental health care settings characterized by intense emotional interactions, high empathic demands, and an elevated risk of burnout (Cleary et al. 2023; Wainberg et al. 2017; Yao et al. 2021). Such challenging care environments can adversely affect nurses' physical and psychological health, overall well‐being, professional practice, and workforce retention (Foster et al. 2020, 2021). However, previous studies have shown that many nurses engage in unhealthy behaviours, such as inadequate sleep, irregular eating patterns, smoking, excessive caffeine consumption, and physical inactivity (Heidke et al. 2020; Reed et al. 2018). Studies have shown that factors such as heavy workloads, feelings of being undervalued within team relationships, and conflicts between workload and the desire to provide optimal care negatively affect psychiatric nurses, while stressful work environments hinder engagement in self‐care practices (O'Malley et al. 2024). Key factors contributing to inadequate self‐care include structural factors such as lack of time and energy, absence of resources for healthy eating and exercise, work‐related challenges, low self‐care awareness, and negative influences from colleagues and leaders (Ross et al. 2019; Bay et al. 2025).

Research on nurses' health indicates that self‐care practices, including healthy nutrition, regular physical activity, and mindfulness‐based interventions, positively influence physical and mental well‐being (Firth et al. 2020; Wexler and Schellinger 2023). Self‐care has been emphasized as a key contributor to the well‐being of mental health professionals, encompassing mindfulness, balance, resilience, physical health, social support, and spirituality, and is recommended for integration into clinical training programs and quality assurance processes (Posluns and Gall 2019). Despite its documented benefits, self‐care is often insufficiently integrated into nurses' daily lives, resulting in adverse consequences for both individual well‐being and professional functioning (O'Malley et al. 2024). Studies among nurses show that self‐care is related not only to nurses' well‐being but also to the caregiving behaviors they provide (Linton and Koonmen 2020; Takmak and Karaçar 2025). Therefore, understanding and supporting self‐care practices among psychiatric nurses is crucial.

Self‐care is defined as an individual's capacity to manage personal health and well‐being through practices such as self‐awareness, self‐regulation, and autonomy (Martínez et al. 2021). Accordingly, individuals consciously engage in self‐care behaviours and participate in health‐related decisions to maintain and improve their physical and mental health (Dorociak et al. 2017; Martínez et al. 2021). This study adopted a theoretical framework to holistically examine the self‐care experiences of psychiatric nurses, considering both behavioural and psychological dimensions. Orem's Self‐Care Deficit Theory emphasizes the importance of individuals recognizing their own care needs and planning behaviours to meet those needs. This allows nurses to maintain their physical, psychological, and social well‐being (Orem 2001). Pender's Health Promotion Model shows that intrinsic motivation and environmental support influence healthy behaviours, allowing for the examination of factors such as workload, team support, and clinical settings as conditions that facilitate or hinder self‐care practices (Pender et al. 2006). Watson's Human Caring Theory emphasizes that the nurse is not only a caregiver but also a care recipient, and by considering self‐care as an ethical and human responsibility, nurses can improve both their own well‐being and the quality of care they provide (Sitzman 2017; Sitzman and Watson 2014). This perspective highlights the central role of nurses' self‐care in supporting their personal well‐being, professional integrity, and quality of care.

In summary, the self‐care experiences of psychiatric nurses need to be addressed and explored, as they are critically important both personally and professionally. Although the importance of nurses' self‐care is well documented (Bay et al. 2025), studies exploring the lived experiences and perspectives of psychiatric nurses remain limited (Gantt and Haberstroh 2023; O'Malley et al. 2024; Saestad Beyene et al. 2025). This study, therefore, aims to explore psychiatric nurses' self‐care experiences and to examine their influence on both individual and professional well‐being. The originality of this study lies in its qualitative examination of nurses' self‐care experiences and in‐depth exploration of the personal, professional, and organizational dimensions. This approach offers a solid foundation, both theoretically and practically, and has the potential to guide interventions and policies to support nurses' self‐care in clinical practice. In this study, self‐care was considered as the subjective experiences and practices of psychiatric nurses related to the individual strategies, coping methods, and support resources they develop to maintain and sustain their physical, psychological, and emotional well‐being while fulfilling their professional responsibilities. The findings are expected to contribute to a deeper understanding of psychiatric nurses' self‐care and support their well‐being and the sustainability of high‐quality mental health care (National Academies of Sciences, Engineering, and Medicine 2021).

## 2 Materials and Methods

### 2.1 Design

This study employed a descriptive phenomenological qualitative methodology to explore psychiatric nurses' self‐care experiences. Descriptive phenomenology aims to describe individuals' views and experiences of a phenomenon from their own perspectives (Creswell and Poth 2018). This approach was appropriate because it enabled an in‐depth understanding of psychiatric nurses' subjective self‐care experiences. Data were collected through individual interviews with psychiatric nurses, and their self‐care experiences were examined using a descriptive phenomenological design. The study was guided by the Consolidated Criteria for Reporting Qualitative Research (COREQ) checklist (Tong et al. 2007).

### 2.2 Study Setting

The study was conducted in the psychiatry clinics of a public hospital in Türkiye. The hospital had an approximate capacity of 1500 beds and included separate psychiatric clinics for women and men, each with 25 beds, as well as a child and adolescent psychiatry clinic with 12 beds. The psychiatric clinics were secluded, with controlled entry and exit, locked doors, and standard safety practices to ensure patient security. Care and treatment for individuals with mental illness were provided using a multidisciplinary team approach.

### 2.3 Participants

The study population comprised 35 nurses working in the psychiatric clinics of public hospitals. Criterion sampling, a purposive sampling technique, was employed. The inclusion criteria were as follows: being a nurse directly involved in patient care in a psychiatric clinic, having at least 1 year of psychiatric nursing experience, and being actively employed at the study site. Nurses with neurological or psychiatric conditions affecting their cognitive status and those on leave, sick leave, or long‐term assignments during data collection were excluded. Of the 35 nurses, 25 met the inclusion criteria, of whom 18 volunteered to participate in this study. All the interviews were completed as planned. No participants withdrew after agreeing to participate in the study. Data collection was conducted with these participants and concluded when sufficient depth was achieved to understand the experiential meaning of the phenomenon.

In line with descriptive phenomenological methodology, data collection focused on obtaining rich and meaningful descriptions of participants' lived experiences rather than achieving data saturation (Giorgi et al. 2017; Polit and Beck 2021). Recent literature suggests that saturation in qualitative research may involve achieving sufficient depth of meaning rather than merely identifying recurring themes (Ahmed 2025). Consistent with this perspective, data collection was concluded when the interviews provided sufficient depth to enable a comprehensive understanding of the experiential meaning of the phenomenon.

### 2.4 Data Collection

Data were collected between May and July 2025 through individual, face‐to‐face, in‐depth interviews. Participants first completed a personal information form capturing demographic characteristics and work experience. A semi‐structured interview guide was developed by the research team based on a review of the literature and consultation with two experts. All interviews were audio‐recorded using this guide. Interview durations ranged from 25 to 45 min, with a mean duration of approximately 30 min.

All interviews were conducted by a female researcher (YK), a PhD‐prepared psychiatric nurse with extensive experience in qualitative research. No prior relationship was established between the participants and the researcher before this study. Participants were informed about the researcher's professional background, study objectives, and purpose of the interviews prior to participation. Interviews were carried out in a safe and comfortable environment to facilitate unbiased responses. They were scheduled either during working hours or outside working hours in the institution's interview room to avoid disruption of clinical services. An audio recording was performed with written informed consent. Participants were asked to describe their self‐care practices and experiences in accordance with the interview guide (Table 1).

**TABLE 1:** Semi‐structured interview form.

*Semi‐Structured Interview Questions* *Opening Question*: How would you define the concept of self‐care? ○ *Probing question*: Why is self‐care important for nurses working in a psychiatric clinic? As a nurse working in a psychiatric clinic, do you have any self‐care practices? If so, what are these practices or activities? How does working in a psychiatric clinic affect your self‐care practices? ○ *Probing question*: What conditions make it easier for you to practice self‐care as a nurse working in a psychiatric clinic? ○ *Probing question*: What situations make it more difficult for you to practice self‐care as a nurse working in a psychiatric clinic? *Closing Question*: Based on your own experiences, do you have any suggestions or anything else you would like to add regarding self‐care for your colleagues?

Data collection focused on obtaining rich and detailed descriptions of the participants' lived experiences. The researcher maintained reflexive awareness throughout the interviews to remain open to the participants' meanings. Field notes were taken during and after the interviews to record contextual observations.

### 2.5 Data Analysis

Data were analysed using Colaizzi's (1978) seven‐stage phenomenological analysis method with MAXQDA software. First, interviews were transcribed verbatim, and researchers familiarized themselves with the data by repeatedly listening to recordings and reading transcripts. Second, significant statements related to self‐care experiences were identified. Third, meanings were formulated from these statements, with researchers working independently. Fourth, similar meanings were clustered to generate themes and subthemes, and consensus was achieved through comparison of independently analysed documents. Throughout the analysis process, reflexive discussions were conducted among the research team to critically examine the emerging interpretations and minimize the influence of preconceptions. Fifth, the phenomenon was described based on the themes and subthemes, supported by illustrative participant quotations. Sixth, the fundamental structure of the phenomenon was articulated. Finally, the findings were returned to participants for validation and feedback. The use of Colaizzi's method ensured a comprehensive and systematic analysis of the phenomenon.

### 2.6 Rigour

This study applied the TACT framework (trustworthiness, auditability, credibility, and transferability) to ensure methodological rigor in qualitative research (Daniel 2019). Trustworthiness was supported by the researchers' expertise in qualitative methods, systematic attention to methodological integrity, and the use of direct participant quotations. The research design, data collection, and analysis procedures were described in detail. Auditability was ensured through comprehensive documentation of the research process, peer review of the data, and consultation with an independent faculty member experienced in qualitative research during the development of main themes and subthemes. Data analysis was conducted by researchers trained in qualitative methods, with themes finalized through consensus. Credibility was enhanced by clearly describing researchers' experience related to the research topic, presenting illustrative participant quotations, and corroborating findings across responses using qualitative analysis software. Consistency across themes contributed to the internal validity of the findings. Transferability was addressed by systematically presenting participants' views and recommendations. Translations were completed by an expert fluent in both Turkish and English.

In descriptive phenomenological research, bracketing involves identifying and holding preconceptions in abeyance; however, it cannot be fully achieved. Therefore, researchers aim to minimize the influence of preconceptions through ongoing reflexivity (Giorgi et al. 2017; Polit and Beck 2021). In this study, reflexivity and bracketing were approached as ongoing and iterative processes throughout the research rather than as a one‐time effort to completely set aside preconceptions. The researcher engaged in continuous self‐reflection to identify and critically examine prior assumptions related to the research topic to minimize their potential influence on data collection and analysis. Reflective notes were maintained before and after each interview to document emerging thoughts, reactions, and possible assumptions, thereby supporting openness to the participants' lived experiences. In addition, reflexivity was strengthened through regular discussions with the research team, allowing for questioning of emerging interpretations and consideration of alternative perspectives. Accordingly, in this study, bracketing was understood not as the complete elimination of preconceptions but as a sustained reflexive effort to remain as open as possible to the experiential meanings emerging from participants' accounts throughout the data collection and analysis.

Researchers have the primary responsibility of protecting participants and their data throughout the research process by implementing rigorous procedures and ensuring confidentiality (Lin 2009). In this study, all ethical and data protection procedures were followed carefully. Participants' personal information was kept confidential, data were securely stored and accessible only to authorized researchers, and the research process was conducted with honesty, respect, and sensitivity while establishing trust with the participants. All interviews were conducted with the participants' informed consent and were audio‐recorded. Each participant was assigned a code to ensure confidentiality, and no identifying information was included in the transcript.

### 2.7 Ethical Considerations

Ethical approval was obtained from the Burdur Mehmet Akif Ersoy University Non‐Interventional Clinical Research Ethics Committee (Approval number: 2025/1217; March 5, 2025). Institutional permission was granted by the Antalya Provincial Health Directorate (Approval number: 25/8; March 19, 2025). All participants provided written informed consent and participated voluntarily. The consent process informed participants that interviews would be audio‐recorded, data would be used solely for scientific purposes, confidentiality would be maintained, and withdrawal from the study was possible at any time.

## 3 Results

Face‐to‐face, in‐depth individual interviews were conducted with 18 psychiatric nurses. Eleven participants were women, with ages ranging from 25 to 40 years. Sixteen participants held a bachelor's degree. Overall nursing experience ranged from 1 to 18 years, while experience in psychiatric clinics ranged from 1 to 12 years. Eleven participants were married, five reported having a chronic illness, and none reported a mental illness. Sociodemographic characteristics of the participants are presented in Table 2.

**TABLE 2:** The characteristic of the participants.

Participants	Age (years)	Gender	Marital status	Education level	Income status	Work experience (years)	Working experience in a psychiatric setting (years)	Weekly working hours	Having a chronic illness	Having a mental illness
P1	30	Male	Married	Bachelor's degree	Income equals expenses	1	1	41–60	No	No
P2	40	Female	Married	Bachelor's degree	Income is more than expenses	18	12	40	No	No
P3	39	Female	Single	Bachelor's degree	Income is less than expenses	15	1	41–60	No	No
P4	34	Female	Married	Bachelor's degree	Income equals expenses	12	1.5	41–60	No	No
P5	28	Female	Married	Bachelor's degree	Income is less than expenses	7	7	41–60	No	No
P6	30	Male	Married	Bachelor's degree	Income equals expenses	7	2	41–60	Yes	No
P7	25	Male	Single	Bachelor's degree	Income is more than expenses	1.5	1	41–60	No	No
P8	28	Female	Married	Master's degree	Income equals expenses	5	1	41–60	Yes	No
P9	25	Female	Single	Bachelor's degree	Income is less than expenses	2.5	1	41–60	Yes	No
P10	25	Female	Single	Associate degree	Income is more than expenses	3	1	41–60	Yes	No
P11	25	Male	Married	Bachelor's degree	Income is more than expenses	1.5	1	41–60	No	No
P12	26	Female	Single	Bachelor's degree	Income is more than expenses	2	1	41–60	No	No
P13	25	Female	Single	Bachelor's degree	Income equals expenses	1	1	41–60	No	No
P14	28	Male	Married	Bachelor's degree	Income equals expenses	6	2	41–60	No	No
P15	33	Female	Married	Bachelor's degree	Income equals expenses	7	7	41–60	Yes	No
P16	32	Male	Married	Bachelor's degree	Income is less than expenses	8	5	40	No	No
P17	25	Male	Single	Bachelor's degree	Income equals expenses	3	1	41–60	No	No
P18	25	Female	Married	Bachelor's degree	Income equals expenses	3	1	41–60	No	No

**FIGURE 1:**
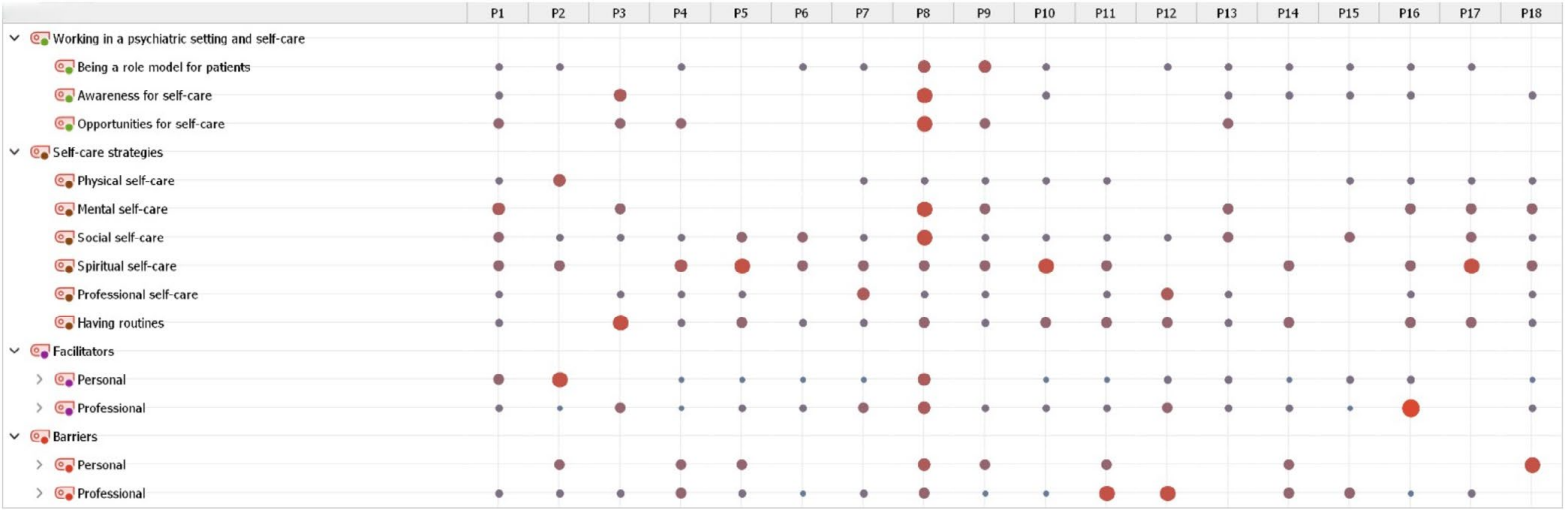
The themes and sub‐themes.

Analysis of the in‐depth interviews yielded four main themes: working in a psychiatric setting and self‐care, self‐care strategies, facilitators, and barriers (Figure 1).

### 3.1 Theme 1: Working in a Psychiatric Setting and Self‐Care

Participants' experiences related to working in a psychiatric setting and self‐care were organized into three subthemes: being a role model for patients, self‐care awareness, and opportunities for self‐care.

#### 3.1.1 Sub‐Theme 1: Being a Role Model for Patients

Participants described the relationship between self‐care and working in psychiatric clinics in terms of their role‐modelling responsibilities. They noted that individuals with psychiatric diagnoses may experience reduced engagement in self‐care. Nurses emphasized that when they attend to their own self‐care, patients are more likely to engage in self‐care behaviours by viewing nurses as role models.

If we are trying to teach them something, we need to be role models. The patient needs to see us as an example so that what we say feels realistic. If I do not take care of myself and my self‐care is inadequate, how can I explain this to the patient? (P1)

#### 3.1.2 Subtheme 2: Awareness for Self‐Care

Participants reported increased awareness of self‐care as a result of working in psychiatric clinics. This awareness was attributed to observing self‐care deficiencies among inpatients, the presence of designated self‐care time in clinics, and learning from patients' experiences.

I think what I learned in the clinic has been very effective in my life. When I'm feeling bad, I do deep‐breathing exercises, focusing on my breath. If I'm very stressed, I try a method like focusing only on that moment and counting numbers or something like that. (P8)

#### 3.1.3 Subtheme 3: Opportunities for Self‐Care

The final subtheme related to working in psychiatric settings concerned opportunities for self‐care. Participants stated that they were able to practice self‐care while caring for patients and that this had a positive effect on their well‐being.

Based on my experience in many different wards, you can devote less time to yourself in other clinics because the focus is mainly on treatment. Here, however, a more holistic approach is possible, and I think that also reflects on us. (P9)

### 3.2 Theme 2: Self‐Care Strategies

Participants were asked about their self‐care practices. Their responses revealed a range of strategies encompassing physical, mental, social, spiritual, and professional dimensions. In addition, participants emphasized the importance of establishing and maintaining routines, as well as engaging in lifelong learning as key self‐care strategies.

#### 3.2.1 Subtheme 1: Physical Self‐Care

Within the physical self‐care subtheme, participants described activities such as walking, swimming, and engaging in sports.

Swimming does me a lot of good. I don't like passive activities done while sitting; movement is important to me. Walking, going to the beach, and taking boat trips really invigorate me. (P2)

#### 3.2.2 Subtheme 2: Mental Self‐Care

Regarding mental self‐care, participants reported using strategies such as mindfulness, recognizing and managing emotions, adopting solution‐oriented approaches, and coping effectively with stress.

Being as solution‐oriented as possible, sharing responsibilities, making a plan, and solving problems step by step, is mentally relieving. (P16)

#### 3.2.3 Subtheme 3: Social Self‐Care

Participants described social self‐care strategies that included seeking social support, applying psychiatric knowledge to social interactions, and using effective communication skills.

After a stressful workday, I go home and talking to my husband feels so good. He's very supportive of me. It's very comforting to be able to communicate and express myself to someone I trust, or someone I think will understand me. (P2)

#### 3.2.4 Subtheme 4: Spiritual Self‐Care

Participants indicated that feelings of gratitude, helping others, and the positive aspects of working as a psychiatric nurse contributed to their spiritual self‐care.

I feel stronger as I realize how much gratitude and positive feedback from my patients makes me happy and how good it feels to help them in some way. (P2)

#### 3.2.5 Subtheme 5: Professional Self‐Care

Professional self‐care was associated with embracing lifelong learning and applying this philosophy in daily practice.

We need to continuously improve ourselves. Even though diagnoses may be similar, each patient is unique, so knowledge is constantly evolving. We have annual training plans, and I provide monthly training with my team to refresh our knowledge and share experiences. (P16)

#### 3.2.6 Subtheme 6: Having Routines

Among self‐care strategies, having routines emerged as one of the most frequently recurring themes. Participants emphasized maintaining regular routines, particularly during stressful periods, as an important self‐care practice.

I make my coffee and watch my favorite series. Even during the busiest shifts, I imagine myself there and tell myself, ‘You'll go home and enter that comfort zone’. Just thinking about it relaxes me. (P13)

### 3.3 Theme 3. Facilitators

The third theme identified in the study was facilitators, which addressed factors influencing psychiatric nurses' ability to maintain self‐care. These factors were categorized into two subthemes, namely personal and professional.

#### 3.3.1 Subtheme 1: Personal Facilitators

Participants indicated that maintaining work‐life balance, having intrinsic motivation, and effective communication were personal factors that facilitated their self‐care experiences.

I try to maintain a work–life balance. I leave what I experience at work behind and go out. I try not to think about it. (P16, work‐life balance)

When I cannot keep up or work alone, I take a deep breath and tell myself, ‘You can do it’. Organizing activities, even later in the evening, has a healing effect on me. (P15, intrinsic motivation)

When dealing with occupational stress, we try to find solutions by sharing with colleagues. Sharing is my main way of coping with stress. (P12, effective communication)

#### 3.3.2 Subtheme 2: Professional Facilitators

Positive workplace experiences and a professional perspective were identified as facilitating factors by the participants.

Getting along well with my teammates makes me feel comfortable. Small, shared moments, like having coffee together, are very valuable, especially because we spend so much time at work. (P9, positive workplace experiences)

I approach situations professionally. I focus on evaluating the patient's condition and what I can do for them, rather than being emotionally affected. (P7, professional perspective)

### 3.4 Theme 4. Barriers

The final theme identified in the study was barriers, which addresses factors negatively influencing psychiatric nurses' ability to maintain self‐care. These factors were categorized into two subthemes, namely personal and professional, each examined in terms of hindering self‐care.

#### 3.4.1 Subtheme 1: Personal Barriers

In the study, the personal barrier cited by the participants was emotional burden. Participants stated that constant communication with patients in a psychiatric setting led to emotional burden and that this was a barrier to self‐care.

Even without psychological distress, this situation causes fatigue and mental strain. Compared with other units, the psychiatry clinic requires constant communication and interaction. Listening to patients' problems, finding solutions, and sharing ideas are mentally exhausting processes. (P18, emotional burden)

#### 3.4.2 Subtheme 2: Professional Barriers

At the professional level, negative workplace experiences, characteristics of psychiatric settings and patients, and shift work were identified as barriers to self‐care.

We constantly struggle to find time for ourselves. Even when patients fall asleep, we cannot leave, and it is difficult to create a space for rest or self‐care. (P17, negative workplace experiences)

The psychiatric ward is highly specialized. There are constant activities, and patients continually expect engagement. You must repeatedly explain things with the same patience, which can be very challenging. (P12, psychiatric patients and settings)

We work 24‐hour shifts, and our eating and sleeping patterns are disrupted. We have little time for ourselves, and even eating under hospital conditions is difficult. (P5, shift work)

### 3.5 The Relationship Between Participant Characteristics and Themes

**FIGURE 2:**
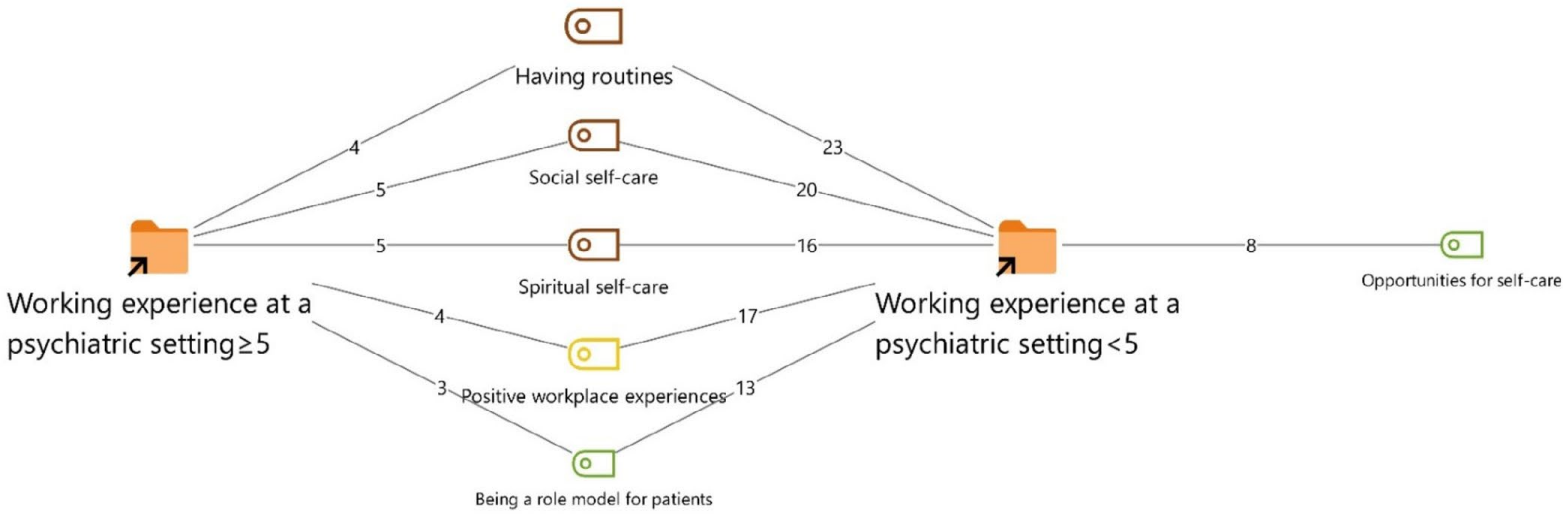
MAXMap for working experience at a psychiatric setting and themes. In the figure, the numbers above the arrows represent the frequency of each theme.

**FIGURE 3:**
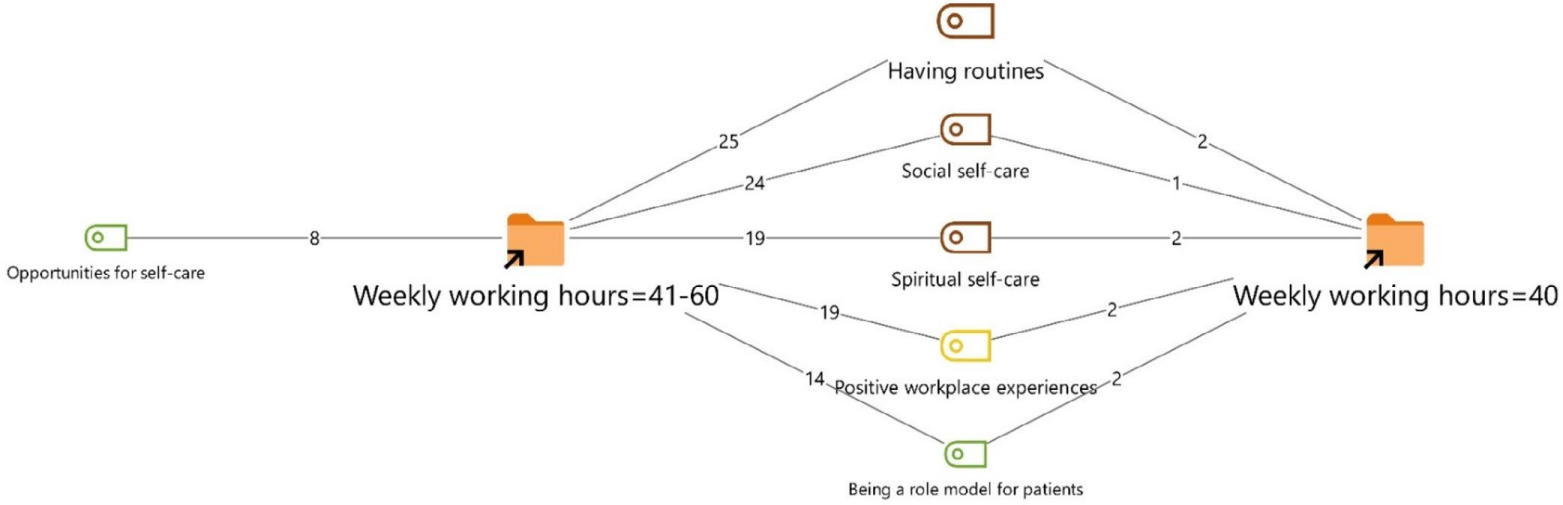
MAXMap for weekly working hours and themes. In the figure, the numbers above the arrows represent the frequency of each theme.

The relationships between the identified themes and participant characteristics, including age, gender, total work experience, psychiatric clinic experience, weekly working hours, and the presence of chronic illness, were examined. Across all characteristics, the most frequently recurring themes were having routines, social self‐care, spiritual self‐care, positive workplace experiences, and being a role model for patients. Participants with less than 5 years of experience in psychiatric clinics and those working 41–60 h per week more frequently described working in a psychiatric clinic as an opportunity for self‐care compared with those with more than 5 years of experience and those working 40 h per week (Figures 2 and 3).

## 4 Discussion

This study is one of the limited number of qualitative studies that have examined the self‐care experiences of psychiatric nurses in depth using a phenomenological approach. Given that the literature mostly focuses on nursing fields outside of psychiatry, this study offers a unique contribution by addressing the self‐care of psychiatric nurses in both individual and professional dimensions and highlights the importance of self‐care for nurses' well‐being and the quality of care.

Analysis of participants' accounts revealed being a role model for patients as a key subtheme under working in a psychiatric setting and self‐care. This finding underscores the importance of self‐care not only for maintaining psychiatric nurses' well‐being but also for their role in modelling health‐promoting behaviours for patients. Nurses have substantial potential to lead change in health care as role models (American Nurses Association 2017). Role modelling in health promotion involves demonstrating healthy behaviours and advocating for well‐being (Darch et al. 2017). Consistent with previous research, nurses' personal health behaviours and attitudes are critical to their effectiveness as role models for patients (Hurley et al. 2018). Júnior et al. (2021) reported that nurses' self‐care behaviours positively influenced team members, encouraging healthier practices. In psychiatric settings, nurses' engagement in self‐care may enhance patients' motivation to adopt self‐care behaviours, thereby supporting the maintenance of care quality. These findings, in line with the theoretical frameworks of Orem, Pender, and Watson, demonstrate that nurses' self‐care is critical for their well‐being and capacity to provide healthy behaviours and quality care to patients (Orem 2001; Pender et al. 2006; Sitzman and Watson 2014).

The findings also indicated that psychiatric nurses developed increased self‐care awareness through their work experiences. This awareness is significant for both nurses' well‐being and the nurse–patient relationship, as it enables nurses to model self‐care behaviours and actively support patients during the care process. Enhanced self‐care awareness may strengthen nurses' well‐being while contributing to improved care quality. Similarly, Kong et al. (2024) reported that nurses' engagement in self‐care and fulfilment of personal needs positively affected the quality of patient care. This awareness, in line with Orem's theory, reinforces the role of nurses in recognizing and meeting their own care needs, while in the context of Pender's model, it highlights the importance of environmental support in translating awareness into behaviour (Orem 2001; Pender et al. 2006). From Watson's theoretical perspective, nurses' self‐care awareness can form a foundation for the sustainability of compassionate care, enhancing both their well‐being and the quality of care they provide to patients (Sitzman and Watson 2014).

The theme of self‐care strategies identified in this study illustrates how psychiatric nurses' self‐care experiences are shaped across physical, mental, social, spiritual, and professional dimensions, as well as through the establishment of daily routines. Participants reported engaging in activities such as walking, swimming, and sports as physical self‐care strategies. Previous research has shown that promoting regular walking positively influences nurses' health behaviours and self‐efficacy (Chaisurin et al. 2023). Similarly, Das and Adams (2021) identified an inverse relationship between physical activity and fatigue, demonstrating that exercise significantly reduces fatigue levels among nurses. These findings suggest that physical activity is a key component of psychiatric nurses' self‐care, supporting occupational functioning by enhancing well‐being and reducing fatigue. These findings support the importance of nurses meeting their own care needs for individual health and occupational functioning (Orem 2001) and the role of intrinsic motivation and environmental support in maintaining healthy behaviour (Pender et al. 2006).

Participants also reported using mental self‐care practices, including mindfulness, recognizing and managing emotions, adopting solution‐oriented approaches, and coping effectively with stress. These strategies are well supported in the literature and are associated with positive outcomes for nurses (Karo et al. 2024; Salem et al. 2025; Wang et al. 2023). Mindfulness practices, in particular, are increasingly used among nurses (Karo et al. 2024). Evidence indicates that nurses who engage in meditation experience fewer depressive symptoms, greater resilience, and lower turnover rates (Wang et al. 2023). Moreover, meditation has been shown to alleviate work‐related distress and fatigue, reduce compassion fatigue, and enhance professional retention (Bonamer and Aquino‐Russell 2019; Wang et al. 2023). Collectively, these findings suggest that mental self‐care practices not only support nurses' psychological well‐being but also contribute to improved professional functioning and care quality.

In this study, nurses adopted social self‐care strategies, such as seeking support, applying effective communication, and transferring psychiatric knowledge to their social lives. These practices indicate that experiences in psychiatric settings foster strong social connections essential for well‐being, quality of life, and professional satisfaction (Fahy and Moran 2018). Social support reduces stress and burnout, improving quality of life and care (Ruisoto et al. 2021; Velando‐Soriano et al. 2020). In spiritual self‐care, the participants emphasized gratitude and helping others. Engagement in spiritual self‐care is linked to greater spiritual well‐being, job satisfaction, and lower stress and burnout (Alazmani‐Noodeh et al. 2021; Ausar et al. 2021). From Watson's perspective, social and spiritual self‐care enhances nurses' self‐compassion and care quality (Sitzman and Watson 2014). Orem's theory shows that meeting psychological and social needs supports personal health and professional sustainability (Orem 2001), while Pender highlights how intrinsic motivation and environmental support sustain these behaviours (Pender et al. 2006).

The adoption of lifelong learning as a professional self‐care strategy emerged as a notable finding in this study. Nurses reported using professional development activities such as planning training programs, updating knowledge, and sharing experiences to support professional growth while also serving a protective self‐care function (Pool et al. 2015). In addition, the use of daily routines, such as drinking coffee, watching television, or imagining personal comfort zones, was identified as an effective self‐care strategy that supports stress management and professional functioning (Wilson et al. 2021). These findings suggest that the deliberate integration of both individual and professional self‐care strategies plays a crucial role in enhancing nurses' well‐being and the quality of care they provide.

The facilitators to nurses' self‐care practices were examined across personal and professional dimensions. Psychiatric nurses identified intrinsic motivation, effective communication, and maintaining work‐life balance as personal facilitators. Bay et al. (2025) reported that encouragement from colleagues and leaders motivates nurses to engage in self‐care, a finding that aligns with the emphasis on intrinsic motivation in the present study. Similarly, effective communication has been associated with increased self‐efficacy and job satisfaction, while work‐life balance contributes to improved subjective well‐being (Ghahremani et al. 2024; Yao et al. 2021). Also, psychiatric nurses in this study identified positive workplace experiences and adopting a professional perspective as professional facilitators of self‐care, indicating that self‐care can be maintained even in demanding psychiatric settings. This underscores the importance of prioritizing self‐care. Moreover, healthy work environments enhance job satisfaction, improve performance, support high‐quality patient care, and contribute to institutional sustainability (Wei et al. 2018). Taken together, this finding, which highlights the role of self‐care in sustaining psychiatric nurses' careers, may inform strategies aimed at improving retention.

In this study, the barriers to nurses' self‐care practices were examined across personal and professional dimensions. Psychiatric nurses identified emotional burden as a personal barrier. Gantt and Haberstroh (2023) suggested that nurses' fatigue and burnout are more closely related to inadequate self‐care than to staffing shortages or workload alone. Similarly, burnout has been shown to negatively affect nurses' mental and physical well‐being (Moss et al. 2016). In the professional sub‐theme, negative workplace experiences, patients' clinical characteristics, and shift work emerged as barriers limiting psychiatric nurses' self‐care practices. A meta‐synthesis study by Bay et al. (2025) identified occupational challenges, such as long working hours, insufficient self‐care awareness, and negative influences from colleagues and managers, as key reasons for nurses' limited engagement in self‐care. Similarly, Dall'Ora et al. (2016) reported that shifts of 12 h or longer were associated with increased error rates and job dissatisfaction. Nurses' difficulty in finding personal time, creating a comfort zone, and continuously monitoring patients clearly illustrates the obstacles to self‐care. When considered collectively, these findings indicate that personal and professional facilitators including intrinsic motivation, effective communication, work‐life balance, positive workplace experiences and professional perspective can promote nurses' self‐care practices, whereas barriers such as emotional burden, negative workplace experiences, demanding patient characteristics, and shift work may hinder this process.

Overall, these findings demonstrate that occupational factors directly influence nurses' self‐care practices, with implications for both nurse well‐being and care quality, emphasizing the need for workplace regulations and conditions that actively support self‐care. These findings, consistent with Orem's theory (Orem 2001), demonstrate that nurses' awareness of personal and professional barriers and the development of strategies to address them are critical for maintaining self‐care among nursing. Within the context of Pender's model, intrinsic motivation and social/environmental support are significant factors influencing nurses' self‐care behaviours (Pender et al. 2006). Watson's theoretical perspective emphasizes that coping with barriers and utilizing facilitators improves the quality of care nurses provide to themselves and their patients (Sitzman and Watson 2014). This theoretical perspective can guide nurses in translating self‐care awareness into behaviour and play an active role in care processes.

Another notable finding of this study is that nurses with less than 5 years of experience in psychiatric clinics more frequently described working in such settings as an opportunity for self‐care than more experienced nurses. Previous research indicates that the early years of nursing are a critical period for the development of self‐confidence and professional identity (Kuokkanen et al. 2016) and that newly qualified nurses actively employ coping strategies such as optimism, self‐efficacy, and seeking social support to manage stress (Han et al. 2022; Tarhan et al. 2023). These findings suggest that early career nurses may be more inclined to interpret their clinical experiences as opportunities for personal and professional development, including self‐care training.

In this study, nurses working longer weekly hours identified working in psychiatric settings as an opportunity for self‐care within their experiences. Increased working hours are associated with greater workload and higher exposure to stress (Kober and Chang 2024), which may in turn heighten the need for self‐care practices. Previous studies have demonstrated that self‐care plays a protective role against burnout and professional fatigue (Lee and Joo 2023), whereas insufficient self‐care is associated with increased emotional exhaustion among nurses (Gantt and Haberstroh 2023). In this context, perceiving the clinical setting as an opportunity for self‐care reflects how nurses, recognizing their self‐care needs in the face of increasing workload and stress, understand and experience self‐care in their coping processes. Taken together, these findings suggest that both professional experience and workload shape nurses' interpretation of their work environment in relation to self‐care. The perception of clinical settings as opportunities for self‐care may contribute to professional resilience and support the sustainability of nursing practices (Alsharif et al. 2024).

### 4.1 Implications for Policy and Practice

Nurses' self‐care awareness and effective use of available opportunities can strengthen both individual and professional well‐being, thereby positively influencing the quality of patient care. This is also essential for the sustainability of the healthcare system, as nurses' ability to maintain their own health and well‐being is vital. In this context, planning and implementing institutional training programs aimed at enhancing nurses' self‐care awareness is important. Integrating topics such as mindfulness‐based stress management, psychological resilience, and self‐care strategies into these programs may support nurses in adopting more conscious and sustainable self‐care practices in daily clinical work. In addition, institutional policies that promote work‐life balance, such as flexible working hours, adequate rest periods, and staff support programs, can further support nurses' professional self‐care.

### 4.2 Limitations

This study has several limitations. Due to its phenomenological design, the sample size was small and focused on in‐depth exploration; therefore, the findings cannot be generalized to all psychiatric nurses. Participants' experiences are subjective. Reflectivity was maintained throughout the study to minimize potential bias. The study was also limited to a single city and institution. Factors such as participants' education level, clinical experience, cultural background, and institutional workload or support systems may have influenced their experiences. Additionally, the limited number of specialist and certified nurses and variation in total work experience may have restricted the representation of the phenomenon. In light of these limitations, future studies should include nurses from diverse geographic and cultural settings, increase sample diversity, and compare experiences across different clinical environments.

## 5 Conclusions

This study highlights psychiatric nurses' self‐care experiences and demonstrates that self‐care is essential for nurses' individual and professional well‐being, as well as for the provision of quality care. The findings show that nurses developed self‐care awareness and adopted diverse self‐care strategies across physical, mental, social, spiritual, and professional domains. However, they also encountered personal and professional barriers, including emotional burden, heavy workload, and negative workplace experiences. Nurses' ability to recognize and use self‐care opportunities can enhance their professional well‐being and, in turn, positively influence the quality of care delivered.

## Author Contributions

All authors contributed to the study conception and design. Sibel Çaynak, Yeliz Karaçar, Buket Şimşek Arslan: conceptualization, methodology, investigation, data curation, writing – original draft, writing – review and editing, visualization. Sibel Çaynak, Yeliz Karaçar, Buket Şimşek Arslan, Ahmet Göktaş, Halil İbrahim Karaçar: conceptualization, methodology, data curation, formal analysis, validation, writing – original draft, supervision, writing – review and editing. All authors read and approved of the final manuscript. All authors have participated sufficiently in the work to take public responsibility for appropriate portions of the content.

## Funding

The authors have nothing to report.

## Ethics Statement

Ethical approval was obtained from the Burdur Mehmet Akif Ersoy University Non‐Interventional Clinical Research Ethics Committee (GO 2025/1217; March 5, 2025). Institutional permission was granted by the Antalya Provincial Health Directorate (25/8; March 19, 2025). All participants provided written informed consent and participated voluntarily. The consent process informed participants that interviews would be audio‐recorded, data would be used solely for scientific purposes, confidentiality would be maintained, and withdrawal from the study was possible at any time.

## Consent

This study was conducted in accordance with the ethical principles outlined in the Declaration of Helsinki. Participants were fully informed, provided with key information about the study that they can consider before deciding whether to take part. All research participants received informed written consent including a detailed explanation of the study.

## Conflicts of Interest

The authors declare no conflicts of interest.

## Data Availability

The data that support the findings of this study are available from the corresponding author, (Yeliz Karaçar) upon reasonable request.
